# Elucidating psychosocial outcome profiles as a function of aggregate genetic risk for suicide attempt and alcohol use disorder

**DOI:** 10.1038/s41398-026-04256-3

**Published:** 2026-07-10

**Authors:** Alexis C. Edwards, Henrik Ohlsson, Mallory Stephenson, Séverine Lannoy, Casey Crump, Jan Sundquist, Kenneth S. Kendler, Kristina Sundquist

**Affiliations:** 1 Virginia Institute for Psychiatric and Behavioral Genetics, Department of Psychiatry, Virginia Commonwealth University, Richmond, VA US; 2 Center for Primary Health Care Research, Lund University, Malmö, Sweden; 3 Department of Family and Community Medicine, McGovern Medical School, The University of Texas Health Science Center, Houston, TX US; 4 Department of Epidemiology, School of Public Health, The University of Texas Health Science Center, Houston, TX US; 5 University Clinic Primary Care, Skåne University Hospital, Malmö, Sweden

**Keywords:** Genetics, Human behaviour

## Abstract

Genetic factors contribute to alcohol use disorder (AUD) and suicide attempt (SA) co-occurrence. Whether individuals with high genetic liability to one outcome versus the other, or to both, differ in terms of their risk for other psychosocial outcomes is unexplored. To this end, the authors conducted a cohort study of Swedish individuals born 1965–1985, with follow-up through December 31, 2018. Using deciles of family genetic risk scores (FGRS), a measure of aggregate genetic liability, they characterized individuals as being in a “risk” group with high/low FGRS or a “reference” with median FGRS. Within each of five comparisons, they calculated correlations between genetic profile and psychosocial outcomes. Groups included up to N = 536 312 individuals. The mean (SD) age at end of follow-up was 44.1 (6.1) years. High genetic liability to both AUD and SA was most prominently correlated with negative outcomes, e.g., *r* = 0.51 for drug use disorder. High FGRS_SA_ or FGRS_AUD_, irrespective of genetic loading for the other, was less strongly correlated with negative outcomes, as was having high FGRS_AUD_ but low FGRS_SA_. The risk subsample with high FGRS_SA_ and low FGRS_AUD_ were least likely to experience negative outcomes (r < 0.1) and more likely to exhibit positive educational outcomes (r≥0.1) relative to their reference subsample. These analyses indicate that aggregate genetic risk profiles for AUD and SA are differentially correlated with a range of negative psychosocial outcomes. Having low genetic liability to AUD is potentially protective for some outcomes even alongside elevated SA risk. These findings underscore the imperative for further genetic dissection of these frequently comorbid conditions.

## Introduction

Suicidal behavior and alcohol use disorder (AUD) frequently co-occur [1–4] and prior evidence indicates that comorbidity is due in part to shared genetic liability [5, 6]. However, their genetic correlation is considerably less than one, i.e., suicidal behavior is influenced to some extent by genetic factors that are unrelated to AUD risk, and vice versa [5]. Although both conditions are genetically correlated with other forms of psychopathology [7–10], little is understood *vis-à-vis* how aggregate genetic liability to both suicidality and AUD, or to only one or the other, is differentially related to other psychosocial outcomes. Further investigation of this question has the potential to improve the identification of risk indicators and predict the sequelae, clinical and otherwise, of these conditions.

Both suicidal behavior and AUD are serious public health concerns. While the incidence of suicidal behavior fluctuates annually and varies by country and sociodemographic features, there were over 49 000 suicide deaths in the US in 2023 [11] and over 720 000 worldwide [12]. Non-fatal suicide attempt (SA) is far more common, with up to 48 attempts for every suicide death in the US in 2022 [13], and SA is one of the most reliable predictors of later death by suicide [14, 15]. In addition to the personal suffering that suicidality entails, there are steep economic consequences: One study estimated that self-harm cost $510 billion annually in the US from 2015–2020 [16] through its impact on medical expenses, reduced quality of life, and other factors.

Similarly, AUD entails a range of intrapersonal, interpersonal, and economic consequences. It is one of the most common psychiatric illnesses, with an estimated 400 million individuals worldwide having a lifetime AUD [17], and harmful alcohol use accounting for 4.7% of the global disease burden in 2019 [18]. The prevalence of alcohol problems and harmful use are highest in high and upper-middle income regions [19]. In the US, excessive drinking cost $249 billion in 2010 due to costs for health care, productivity loss, and other expenses [20].

In the current study, we examine whether genetic liability to suicidality or AUD, or to both of these, are differentially related to other psychosocial outcomes. Our research question was motivated by the degree of comorbidity between AUD and SA – one US study found that, among individuals with AUD or SA, 12.2% were comorbid [21] – and the increasingly urgent need to identify indicators of latent risk. The high degree of polygenicity of each condition [9, 10] and their substantial but incomplete genetic correlation [5] will correspond to disparate combinations of relative genetic liability for AUD and/or SA, which may result in qualitatively or quantitatively distinct patterns of psychosocial correlates. Clarifying these profiles could help inform efforts by health care providers to evaluate risk of AUD and/or SA even in the absence of genetic information.

To pursue our research aims, we use a genetically informative Swedish birth cohort to classify individuals based on their aggregate genetic liability for SA and AUD, and to calculate correlations between these genetic profiles and a range of psychosocial outcomes. These include educational measures, marital status, psychopathology, and receipt of social benefits (e.g., unemployment) that are indicators of financial precarity. Our primary aims are: (i) to clarify how genetic liability underlying SA and/or AUD could manifest outside the context of these conditions themselves, and (ii) to consider whether distinct genetic risk profiles – e.g., high genetic liability to SA but not AUD, or vice versa – are differentially related to psychosocial outcomes, some of which could be considered risk indicators or opportunities for prevention/intervention. Prior studies suggest that the extent to which genetic factors contribute to suicidal behavior and/or AUD is comparable between Sweden and other high-income countries [5, 22–24]. Detailed national registry data on conditions and exposures relevant to public health outcomes alongside the genetically informative nature of this population enables us to address these research questions in a manner not possible in other contexts.

## Methods

### Sample

We collected information on individuals from Swedish population-based registers with national coverage linking each person’s unique personal identification number (which was replaced with a serial number by Statistics Sweden to preserve confidentiality). Ethical approval for this study was granted by the regional ethical review board in Lund (No. 2008/409 and later amendments). Participant consent was not required. Our database consisted of all individuals born in Sweden between 1965 and 1985 (N = 1 798 912) to Swedish parents (to ensure sufficient power for genetic risk calculation) followed up through 12-31-2018, with a mean (SD) age at end of follow-up of 43.9 (9.1).

### Data resources

We used data from a range of national registers as described in detail in the Supplementary Table 1. We also used information from Primary Care. This is a research dataset including individual-level almost nationwide information on clinical diagnoses from primary health care centers [25]. These resources enable us to identify relatives as needed to generate Family Genetic Risk Scores (FGRS; see below). They further provide information on the exposure and outcome variables (described below) through the use of medical, criminal, educational, health insurance, and military records. At the end of the follow-up period, the registers cover nearly 100% of the Swedish population.

### Exposure variable definition

The exposure was a function of aggregate genetic risk for SA or AUD, operationalized as FGRS (FGRS_SA_ and FGRS_AUD_). The FGRS are calculated from morbidity risks for disorders in 1st–5th degree relatives, controlling for cohabitation effects (see Supplementary Table 2). The scores, which adjust for cohabitation, are based on phenotypes in extended relatives rather that from molecular data, and have been shown to reflect components of genetic liability that are complementary to polygenic scores based on molecular genetic data [26]. FGRS are standardized as described in Supplementary Table 2.

**Fig. 1:**
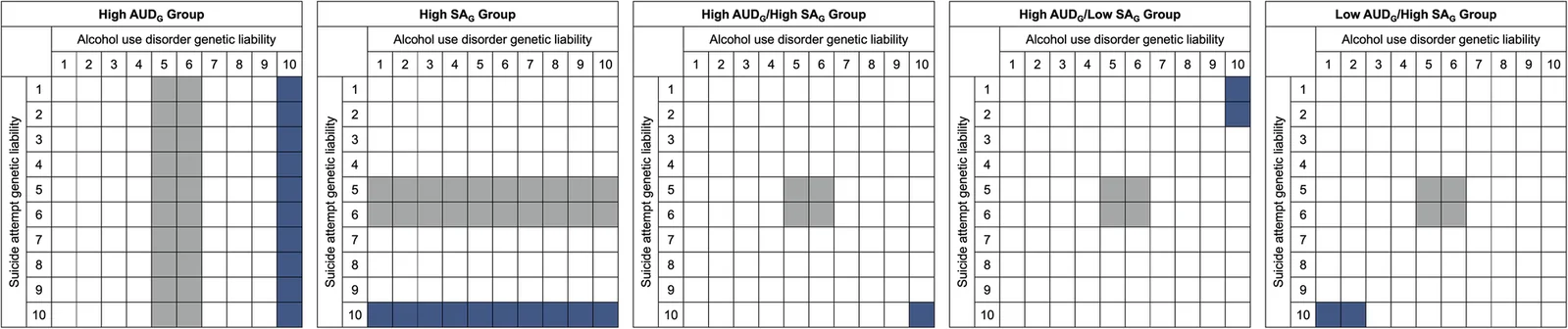
Depiction of genetic profiles of risk (in blue) and reference (in grey) subsamples for each analytic group. Genetic liability was operationalized as a family genetic risk score, which was then categorized into deciles.

Similar to a previous study [27], we divided the total cohort (of nearly 1.8 million) based on FGRS_AUD_ and/or FGRS_SA_. In each of five groups, “high” genetic risk was operationalized as being in the highest FGRS decile for the outcome(s) of interest; “median” genetic risk reflected being in the middle two deciles; and “low” genetic risk corresponded to being in the lowest one or two FGRS deciles (depending on group sample size). In each group, the reference subsample had median FGRS for AUD and/or SA. A visual depiction of the genetic profile of each group is provided in Fig. 1, and groups are named based on the genetic profile of the subsample of interest. The first group (N = 536 160) consisted of individuals with high versus median FGRS_AUD_, irrespective of FGRS_SA_; it is termed “High AUD_G_”, where the “G” denotes genetic liability. The second group (N = 536 312), called “High SA_G_”, included individuals with high versus median FGRS_SA_, irrespective of FGRS_AUD_. The “High AUD_G_/High SA_G_” group (N = 179 639) compared those with high FGRS_SA_ and FGRS_AUD_ versus those with median risk for both. The fourth group (N = 151 153), “High AUD_G_/Low SA_G_”, consisted of individuals with *high* FGRS_AUD_ and *low* FGRS_SA_, compared to those at median risk for both. Individuals in the final group (N = 152 865), “Low AUD_G_/High SA_G_”, had *low* FGRS_AUD_ and *high* FGRS_SA_, compared to those at median risk for both. For simplicity, we will refer to individuals in each group with the “median” FGRS as the reference subsample, and those selected for high/low FGRS as the risk subsample. We note that the reference subsample is not identical across groups: Comparisons across groups are conceptual rather than technical. The first two groups essentially serve as baseline conditions for comparison to the remaining groups, whose profiles are of particular interest. Sample sizes for each group are provided in Table 1.

**Table 1:** Sample sizes and prevalence of psychiatric and social outcomes for risk and reference subsamples in each of five selected groups.

	High AUD_G_	High SA_G_	High AUD_G_/High SA_G_	High AUD_G_/Low SA_G_	Low AUD_G_/High SA_G_
	High versus median genetic risk for AUD	High versus median genetic risk for SA	High genetic risk for both AUD and SA to those at median risk for both disorders	High genetic risk for AUD and low genetic risk for SA to those at median risk for both disorders	High genetic risk for SA and low genetic risk for AUD to those at median risk for both disorders
	Risk/Reference	Risk/Reference	Risk/Reference	Risk/Reference	Risk/Reference
N	178 558/357 602	178 679/357 633	54 584/125 055	26 098/125 055	27 810/125 055
Mean Year of Birth	1975/1974	1975/1973	1975/1972	1976/1972	1976/1972
Females	49.1%/48.5%	49.7%/48.1%	49.7%/48.0%	48.7%/48.0%	49.3%/48.0%
Alcohol use disorder (AUD)	11.7%/3.5%	8.9%/4.1%	15.3%/3.3%	7.0%/3.3%	3.4%/3.3%
Suicide attempt (SA)	7.4%/3.2%	8.2%/2.9%	11.0%/2.5%	4.3%/2.5%	5.1%/2.5%
Academic achievement (mean)	−0.36/0.04	−0.24/0.01	−0.55/0.03	−0.02/0.03	0.22/0.03
N (Academic achievement)	111 084/203,655	111 799/183,451	34 071/58 881	18 950/58 881	19 654/58 881
Educational attainment (EA)	3.03/3.42	3.17/3.38	2.84/3.38	3.43/3.38	3.69/3.38
Divorce	8.7%/6.0%	8.3%/6.0%	10.0%/5.7%	6.6%/5.7%	6.0%/5.7%
N (Divorce)	92 329/205 923	97 687/ 207 169	27 995/ 74 095	13 099/ 74 095	15 121/74 095
Married	51.7%/57.6%	54.6%/57.9%	51.2%/59.2%	50.2%/59.2%	54.4%/59.2%
Anxiety disorders	24.8%/15.5%	23.2%/15.4%	29.2%/14.1%	20.8%/14.1%	17.6%/14.1%
Attention deficit hyperactivity disorder	4.8%/1.3%	3.3%/1.3%	5.3%/1.1%	2.4%/1.1%	1.5%/1.1%
Bipolar Disorder	2.6%/1.3%	2.5%/1.2%	3.5%/1.1%	1.8%/1.1%	1.6%/1.1%
Drug use disorder	10.1%/2.8%	8.1%/3.0%	13.9%/2.3%	5.8%/2.3%	3.2%/2.3%
Major depression	24.4%/16.0%	23.6%/15.8%	28.6%/14.9%	20.3%/14.9%	17.7%/14.9%
Posttraumatic stress disorder	18.1%/12.7%	17.8%/12.8%	20.9%/12.2%	14.6%/12.2%	13.7%/12.2%
Resilience (mean)	3.62/4.13	3.79/4.13	3.39/4.16	3.90/4.16	4.21/4.16
N (resilience)	74 493/158 556	73 955/161 699	21 830/57 180	11 004/57 180	11 784/57 180
Area Deprivation					
1 year	7.9%/6.6%	7.5%/6.7%	6.6%/6.6%	6.6%/6.6%	6.0%/6.6%
>1 year	19.5%/14.3%	18.3%/14.6%	22.3%/14.2%	15.0%/14.2%	12.9%/14.2%
Early Retirement	4.6%/2.4%	4.4%/2.4%	6.1%/2.2%	3.0%/2.2%	2.8%/2.2%
Sick Leave					
1year	18.7%/18.3%	18.8%/18.2%	19.0%/18.4%	17.7%/18.4%	17.5%/18.4%
>1 year	20.9%/16.2%	20.6%/16.1%	23.6%/15.8%	16.4%/15.8%	15.5%/15.8%
Social Welfare					
1 year	4.7%/2.2%	4.1%/2.3%	5.8%/2.1%	3.0%/2.1%	2.0%/2.1%
>1 year	8.8%/2.8%	7.4%/3.0%	12.5%/2.6%	4.2%/2.6%	2.4%/2.6%
Unemployment					
1 year	11.8%/10.1%	11.9%/10.2%	12.3%/10.0%	10.6%/10.0%	9.2%/10.0%
>1 year	20.7%/14.9%	18.9%/15.6%	23.6%/15.5%	15.5%/15.5%	12.6%/15.5%

### Outcome variable definition

In the database we also include four domains of outcome variables. Educational outcomes were operationalized by academic achievement at age 16 (AA) and educational attainment (EA); Family outcomes were captured by registrations for marriage (MAR) and divorce (DIV); Psychiatric/Psychological outcomes were based on lifetime registrations of anxiety disorders (AD), attention deficit hyperactivity disorder (ADHD), bipolar disorder (BD), drug use disorder (DUD), major depression (MD), posttraumatic stress disorder (PTSD) and a resilience score (available for males only); and Socioeconomic outcomes were registrations for receipt of social welfare (SW), unemployment (UE), early retirement (ER), residing in deprived area (DEP) and sick leave (SL). Supplementary Table 1 provides details for all variables.

### Statistical analyses

Within each sample, we calculated the tetrachoric/polychoric correlation (for AA we used the biserial correlation as AA was measured on a continuous scale) between the two selected groups (e.g., risk subsamples with high FGRS_SA_ versus reference groups with median FGRS_SA_) for the occurrence of each of the selected outcomes. That is, for example, a two-by-two table from which the correlation calculated would be the number of individuals in the risk subsample without and with a particular disorder and the number of individuals in the reference subsample without and with that same disorder. These correlations provide standardized, prevalence-adjusted associations that are comparable across outcomes with different scales. The method assumes that each observed binary/ordinal variable reflects an underlying latent normally distributed variable with fixed thresholds. Higher correlations would reflect greater differences in risk for that disorder between the two subsamples. We included three regression terms for year of birth, sex, and county of residence on the thresholds. In a sensitivity analysis we excluded all individuals with AUD or SA and replicated all analyses. We used Mplus software version 7.31 [28] and SAS 9.4 [29] software for all analyses. Error bars represent 95% confidence intervals and we relied on these, rather than *p*-values, for statistical inference.

## Results

### Descriptive statistics

Table 1 presents the sample sizes and risk/reference distributions for each analytic group. The number of risk and reference subsamples varied across group: The High AUD_G_ and High SA_G_ groups included ~536 K individuals (including ~100 K at risk), the High AUD_G_/High SA_G_ group consisted of ~189 K individuals ( ~ 33 K at risk), and the High AUD_G_/Low SA_G_ and Low AUD_G_/High SA_G_ groups included ~150 K individuals (with ~23 K at risk). While patterns of poorer outcomes for educational attainment and academic achievement were observed among the risk subsamples, a notable difference was those in the last group – Low AUD_G_/High SA_G_ – exhibited higher levels of both relative to their reference subsample. With respect to family-related outcomes, similar differences were observed. Overall, high risk subsamples were less likely to have been married, and if they had married, were more likely to be divorced.

For each of the six psychiatric outcomes, the prevalence was higher among at-risk subsamples in each, though the magnitude of difference varied considerably. For example, within the High AUD_G_/High SA_G_ group, ADHD was nearly 5 times as common in the at-risk subsample as in the reference subsample, while major depression was approximately twice as common; drug use disorders were 6-fold more common in High AUD_G_/High SA_G_ subsamples versus their reference, but only 1.39-fold more common in Low AUD_G_/High SA_G_ subsamples versus their own reference subsample. At-risk subsamples also exhibited higher rates of financial hardship indicators, such as receipt of social welfare, long-term unemployment, and early retirement (similar to long-term disability in the United States).

### Correlations within outcome category

**Fig. 2:**
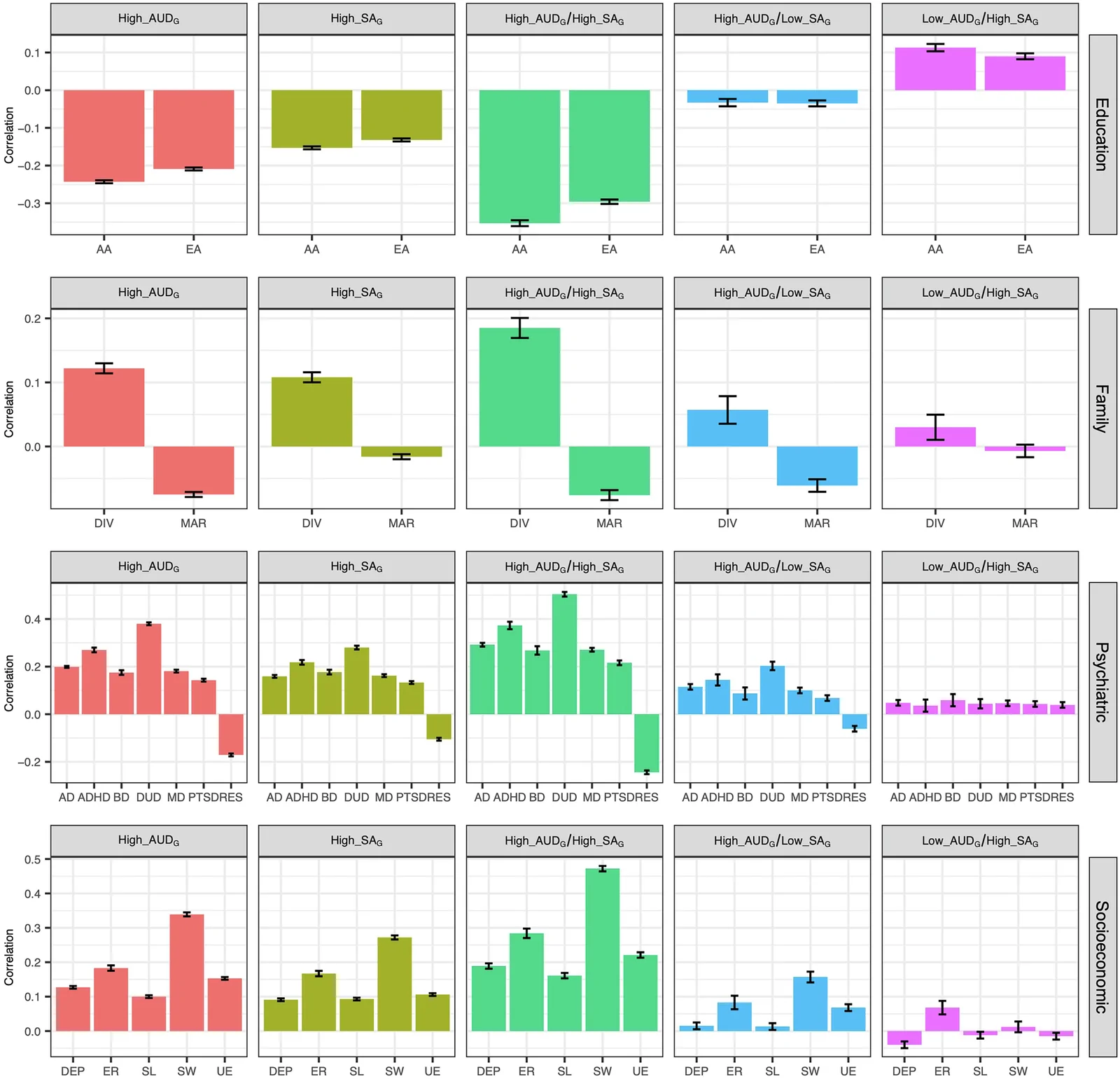
Correlations and 95% confidence intervals between risk/reference status and each psychosocial outcome. Rows represent thematic categories; columns represent each of the five analytic groups. Within row, the y-axis scale is consistent across groups, and the y-axis varies across rows. *Notes for* Fig. 2: AA academic achievement, EA educational attainment, DIV divorce, MAR marriage, AD anxiety disorders, ADHD attention deficit hyperactivity disorder, BD bipolar disorder, DUD drug use disorder, MD major depression, PTSD post-traumatic stress disorder, RES resilience, DEP deprived neighborhood residence, ER early retirement, SL sick leave, SW social welfare, UE unemployment.

Polychoric correlations between risk/reference status and each psychosocial outcome are depicted in Fig. 2. Correlations between both FGRS and each outcome are provided in Supplementary Table 3.

#### Education outcomes

With the exception of the Low AUD_G_/High SA_G_ group, risk subsamples were more likely to have lower levels of academic achievement and educational attainment. The most substantial correlations were within the High AUD_G_/High SA_G_ group (r = −0.30 to −0.35), while in other cases the correlations were quite small and negative (e.g., r = −0.04 to −0.03 in Group 4). In the Low AUD_G_/High SA_G_ group, the magnitude of correlations with these outcomes were also small but positive (r = 0.09–0.11).

#### Family outcomes

For the first four groups, risk status was slightly inversely correlated with having ever been married (r’s ranged from −0.08 to −0.06); for the Low AUD_G_/High SA_G_ group, the correlation was negative but did not differ from 0. Among individuals who had been married, correlations with divorce were positive and significant, but small (0.03 to 0.12), across all groups.

#### Psychiatric/psychological outcomes

In general, the strongest correlations between risk/reference status and psychiatric outcomes were observed in the High AUD_G_/High SA_G_ group, within which the at-risk subsample had high genetic liability for both AUD and SA. Correlations in the first two groups (High AUD_G_ and High SA_G_) were slightly lower; estimates for the High AUD_G_/Low SA_G_ group were lower still; and correlations within the Low AUD_G_/High SA_G_ group for these outcomes were lowest, ranging from only 0.04 to 0.05. The correlation between High AUD_G_/High SA_G_ risk/reference status and DUD was the strongest observed across all categories and groups (r = 0.51 [0.50; 0.51]). For the first four groups, the highest magnitude correlation within psychiatric outcomes was for DUD; furthermore, relatively strong correlations were also observed for ADHD, the other primary externalizing disorder. Correlations with the internalizing disorders (MD, AD, and PTSD) were lower overall within these groups. In contrast, the Low AUD_G_/High SA_G_ group was the only one whose highest correlations were with AD and MD, though these were weak. This was also the only group where being at-risk was positively associated with resilience.

#### Socioeconomic outcomes

The strongest correlations between risk/reference status and adverse socioeconomic outcomes were observed in the High AUD_G_/High SA_G_ group, where they ranged from 0.16–0.47. For the first four groups (excluding Low AUD_G_/High SA_G_), the highest magnitude correlation within socioeconomic outcomes was for social welfare receipt. Correlation patterns were quite similar across those four groups, though the magnitude of effect varied. In contrast, correlations between Low AUD_G_/High SA_G_ risk/reference status and socioeconomic outcomes were consistently very low. For sick leave, neighborhood deprivation, and unemployment, the correlations were negative (i.e., individuals in the reference group were more likely to have experienced these adverse socioeconomic conditions); the correlation with social welfare receipt was not significant; and a positive correlation was observed only for early retirement (r = 0.07 [0.05; 0.09]).

### Secondary analyses

We pursued a sensitivity analysis in which we excluded all individuals with AUD and SA to determine whether our initial findings were driven by this subgroup. The resulting correlations, depicted in Supplementary Fig. 1, shifted modestly in magnitude but did not substantively differ.

Finally, to disambiguate whether findings from the Low AUD_G_/High SA_G_ group are due to the potentially protective status of having *low* FGRS_AUD_ versus *high* FGRS_SA_, we conducted a *post hoc* analysis in which one subsample had low FGRS_AUD_ but median FGRS_SA_, while the reference subsample had median FGRS for both AUD and SA. The test subsample in this case has a lower genetic burden than the reference subsample, hence we do not refer to it as a “risk” subsample. A direct comparison with the Low AUD_G_/High SA_G_ group is depicted in Supplementary Fig. 2. For educational outcomes, we observed small positive correlations with test/reference status, with point estimates exceeding those for the Low AUD_G_/High SA_G_ group. We observed a small inverse correlation with divorce, i.e., the subsample with low FGRS_AUD_ were less likely to get divorced than the reference subsample. Likewise, test/reference status was weakly but significantly inversely correlated with all the examined psychiatric diagnoses, with the exception of bipolar disorder, for which confidence intervals spanned 0; the correlation with resilience was weakly positive. Finally, we observed weak but significant inverse correlations with all adverse socioeconomic outcomes other than early retirement.

## Discussion

In the current study, we aimed to characterize psychosocial profiles among individuals who were classified according to their relative aggregate genetic risk for alcohol use disorder and suicide attempt. These outcomes often co-occur, and previous studies have found evidence that the co-occurrence is likely attributable to both shared genetic liability [30] and a causal pathway [31]. Key findings were as follows: (i) Being at high genetic risk of both AUD and SA, relative to median risk for both (i.e., High AUD_G_/High SA_G_), was consistently most strongly correlated with poorer outcomes across educational, family, psychiatric, and socioeconomic categories; (ii) individuals at high genetic risk for AUD *or* SA were less strongly, but still consistently, associated with adverse outcomes, with those in the former group faring more poorly; (iii) within the considerably smaller High AUD_G_/Low SA_G_ group, patterns of correlations overall resembled those in the first three groups, though at generally lower magnitudes; and (iv) the Low AUD_G_/High SA_G_ group exhibited considerably different correlation patterns, such that correlations with all categories of outcomes were quite low, and risk subsamples were likely to have better educational outcomes than reference groups. Thus, considering bivariate family history-based genetic liabilities could provide potentially informative insight to risk profiles for a wide range of psychosocial outcomes.

The fact that individuals with a high FGRS family history of both AUD and SA are most likely to experience adverse outcomes is common sensical, but our findings add empirical support to this intuition, demonstrating that such individuals likely warrant particular attention. Notably, being in the risk subsample in any of the first three groups was correlated with adverse outcomes even early in life – namely, in risk subsamples’ lower academic achievement at age 16 and lower overall educational attainment (though the latter could stretch into adulthood). This could reflect prodromal difficulties related to AUD/SA. In addition, prior studies have revealed a complex association between educational/cognitive factors and AUD [32–36] and SA [37, 38], including evidence of a protective causal effect of academic achievement and educational attainment [39–41]. The beneficial causal impact of educational measures extends to other psychiatric and substance use outcomes [41–43]. Thus, early interventions that improve educational outcomes might actually reduce the need for subsequent increased resource allocation.

An unexpected feature of our findings is that being in the at-risk subsample within the Low AUD_G_/High SA_G_ group was correlated with several advantageous outcomes (educational measures, resilience, lower likelihood of living in a deprived neighborhood, and lower likelihood of >1 year of unemployment or sick leave). However, those correlations – alongside the associations with adverse outcomes – were quite weak. A conservative interpretation of these results is that these persons are generally quite comparable to reference groups with respect to psychosocial outcomes. This contrasts slightly to risk subsamples in the High AUD_G_/Low SA_G_ group, who bear the complementary genetic burden. The profile of that group presents as a less severely affected version of the first three, and we did not observe correlations with advantageous outcomes. However, given the attenuation of positive correlations with negative outcomes relative to the first three groups, it is reasonable to conclude that having low genetic liability to SA does confer some degree of protection against adverse outcomes, though to a lesser degree than having low genetic liability to AUD. Future studies could perhaps further dissect the findings from the High AUD_G_/Low SA_G_ and Low AUD_G_/High SA_G_ groups, particularly taking into consideration prior evidence that a non-trivial proportion of suicide deaths [44] and attempts [45] occur among individuals with no known history of psychiatric illness who may otherwise be high-functioning.

Given the patterns of results across these groups, we considered the possibility that having low genetic liability for AUD – which applied only to the at-risk subsample of the Low AUD_G_/High SA_G_ group in the primary analyses – conveys a general protective effect against adverse psychosocial outcomes that largely outweighs the potential negative consequences of having high genetic liability to SA. The inverse is less well supported given positive correlations with adverse outcomes in the High AUD_G_/Low SA_G_ group. Furthermore, a scenario in which high FGRS_SA_ is somehow protective is not supported by results within the High SA_G_ group, where the risk subsample has high FGRS_SA_ but still experience poorer outcomes. Both SA and AUD have been genetically and phenotypically associated with various externalizing behaviors and impulsivity [46–50], which in turn are associated with higher rates of social dysfunction [51, 52]. Our findings indicate that the risk subsample in the Low AUD_G_/High SA_G_ group are considerably less likely to exhibit these outcomes than risk subsamples in other groups. Yet the possibility that the at-risk subsample of the Low AUD_G_/High SA_G_, relative to those in the High AUD_G_/Low SA_G_ group, simply represent individuals with a more “internalizing” phenotype is not borne out by the results: Correlations with major depression, anxiety disorders, and PTSD were only weakly positive, and were lower than for the latter group.

Our *post hoc* analysis aimed to disambiguate the potentially protective role of low FGRS_AUD_ versus high FGRS_SA_. Given that the only significant correlations with risk/reference status were positive for advantageous outcomes (e.g., better educational outcomes) and negative for adverse outcomes (e.g., divorce, several psychiatric disorders), we infer that low FGRS_AUD_ is the driving force underlying the initially unexpected Low AUD_G_/High SA_G_ results. As with our other findings, replication in an independent sample is warranted. In the interim, clinicians who learn of a patient’s dense family history of suicidality should still consider those individuals to be at elevated risk – even in circumstances where they enjoy elevated social capital or exhibit few other risk indicators – as the risk subsample in the Low AUD_G_/High SA_G_ group had poorer outcomes relative to our *post hoc* group.

A number of extensions of the current approach can be pursued in samples for which molecular data are available. Polygenic scores for AUD and SA, divided into deciles, can be used to assign risk/reference status and evaluate whether phenotypic outcome profiles reflect similar patterns as reported in the current study. In addition, a multivariate GWAS could identify specific variants that are associated with increased risk of SA but decreased risk of AUD (or vice versa); secondary analyses (e.g., gene-based and tissue enrichment analyses) could be used to further clarify the biological etiology of SA or AUD in this narrow context. Although the at-risk subsample in the Low AUD_G_/High SA_G_ group did exhibit some minor elevations in risk of adverse outcomes (e.g., divorce, early retirement, and psychiatric disorders), in other respects they could be considered relatively high-functioning (e.g., higher academic achievement and educational attainment, lower likelihood of living in a deprived neighborhood) and therefore of potential interest for additional research on resilience.

We note several limitations to the current study. First, our estimates of aggregate genetic risk for AUD and SA are derived from family-based data rather than molecular data. The scores are adjusted for a wide range of potential factors including cohabitation; furthermore, they have been compared to genotype-based approaches and found to reflect the same underlying liability dimension for psychiatric illnesses [26, 53]. However, replication in samples for which molecular data is available would bolster confidence in our findings.

Second, although FGRS are adjusted for cohabitation by family members, they do not account for environmental exposures unshared by family members that may jointly increase AUD and SA risk. Prior research suggests that such factors contribute to AUD and SA comorbidity [5]. Identification of those exposures – some of which might be among the outcomes explored here – and whether they exacerbate the relative impact of genetic liability should be the topic of future research efforts.

Third, our approach relies on Swedish national registry data. This has the advantage of nationwide coverage, consistent measurement, and avoidance of recall bias. However, the registrations that were used to derive each FGRS, and the registrations for psychiatric outcomes, reflect conditions that were serious enough to warrant medical attention. Because many individuals with AUD do not seek treatment or otherwise escape detection by health care professionals [54], and in the US only a slim majority of suicide attempts require medical attention [55], we are likely capturing mostly severe manifestations of these conditions. Were FGRS estimations to include family members who, for example, engaged in only less serious suicide attempts, FGRS profiles could shift along with corresponding associations with outcomes. Relatedly, more extensive observation periods could lead to shifts in the number of individuals in FGRS-based risk and/or reference subsamples with registrations for psychiatric registrations or marriage/divorce, which could impact the magnitude of observed correlations. However, since the mean birth year of individuals included in the analyses ranges from 1972–1976, we would expect subsequent psychiatric registrations to be minimal.

Fourth, Sweden has a robust social safety net apparatus and universal health care. In countries where such services are harder to access, the magnitude of correlations with risk/reference status across groups might differ from those reported here. Finally, FGRS derivation is dependent on the availability of sufficient health care records from family members; we are thus limited to conducting analyses on individuals with Swedish-born parents, which excludes Sweden’s considerable foreign-born population [56].

In summary, the current findings demonstrate the wide-ranging psychosocial consequences of having a high genetic liability for AUD and/or SA, while also illuminating the nuanced nature of these correlations. As expected, having high genetic liability to both conditions was most strongly correlated with adverse educational, familial, psychiatric, and socioeconomic outcomes, while correlations were more modest for those with elevated genetic risk for only AUD or SA. One of the more compelling observations was that individuals at high genetic risk for SA but low genetic risk for AUD exhibited only minor correlations with most adverse outcomes, alongside weak correlations with several positive outcomes; this raises questions regarding how genetic risk that is specific to SA – to the exclusion of that for AUD, a commonly comorbid condition – is unique.

## Supplementary information

Supplementary Material

## Data Availability

Data access is restricted and is available only through application to Statistics Sweden.
