# Supplementary material for: Elucidating psychosocial outcome profiles as a function of aggregate genetic risk for suicide attempt and alcohol use disorder
Source: Transl Psychiatry. 2026 Jul 10;16:519. doi: 10.1038/s41398-026-04256-3 (PMC13639089; doi:10.1038/s41398-026-04256-3)
Supplement: Supplementary file 1 [file 41398_2026_4256_MOESM1_ESM.pdf]

Supplemental Material

Elucidating psychosocial outcome profiles as a function of aggregate genetic risk for suicide attempt and alcohol use disorder

Alexis C. Edwards, PhD, Henrik Ohlsson, PhD, Mallory Stephenson, PhD,
Séverine Lannoy, PhD, Casey Crump, MD, PhD, Jan Sundquist, MD, PhD,
Kenneth S. Kendler, MD, Kristina Sundquist, MD, PhD

Supplemental Methods

Data resources. Detailed descriptions of several registries used in the current analyses are available online:

- Multi-Generation Register. See: https://www.scb.se/en/finding-statistics/statistics-by-subject-area/other/other/other-publications-non-statistical/pong/publications/multi-generation-register-2016/
- Register of the total population. See: https://www.scb.se/contentassets/8f66bcf5abc34d0b98afa4fcbfc0e060/rtb-bar-2016-eng.pdf
- National Patient Register. See: https://www.socialstyrelsen.se/en/statistics-and-data/registers/national-patient-register/

Supplementary Table 1 details which registers were used for each variable in the current analyses. Supplementary Table 2 details the method used to calculate family genetic risk scores (FGRS).

Supplementary Table 1. Description of register and other resources used in the current analyses.

	Registers Used	Definition
Suicide Attempt (SA)	The Swedish Hospital Discharge Register (coverage 1973-2018); Outpatient Care Register (national coverage 2001-2018); Primary Care Registry (Partly coverage from 1999-2018)	ICD-9: E950-E959 and E980-E989<br>ICD-10: X60-X84 and Y10-Y34<br><br>Registrations that were follow by a suicide death within 30 days were not counted.
Alcohol Use Disorder (AUD)	The Swedish Hospital Discharge Register (coverage 1973-2018); Outpatient Care Register (national coverage 2001-2018); Primary Care Registry (Partly coverage from 1999-2018); the Swedish Drug Register (2005-2018); the Swedish Mortality Register, and the Swedish Criminal Register (1973-2018) and the Swedish Suspicion Register (1998-2018)	Alcohol Use Disorder (AUD) was identified in the Swedish medical and mortality registries by ICD codes: ICD-9: V79B, 305A, 357F, 571A-D, 425F, 535D, 291, 303, 980; ICD-10: E244, G312, G621, G721, I426, K292, K70, K852, K860, O354, T51, F10); in the Swedish Criminal Register and the Swedish Suspicion Register with at least two registrations of drunk driving (suspicion code 3005, law 1951:649 (paragraph 4 and 4A)) or drunk in charge of a maritime vessel (suspicion code 3201, law 1994:1009 (chapter 20, paragraph 4 and 5)); in the Prescribed Drug Register by the drugs disulfiram (Anatomical Therapeutic Chemical (ATC) Classification System N07BB01), acamprosate (N07BB03), and naltrexone (N07BB04).
Academic achievement (AA)	National School Register	Contains educational achievement (a grade point average) for all students at the end of basic high school (grade nine; usually at age 16). Students had an incentive to perform well in this school year because those with high grades were more likely to gain admission to the desirable upper secondary schools. For each year and by gender we standardized the grade score into a Z-score. From 1988 to 1997, the score was expressed on a scale between 1 (lowest) and 5 (overall mean was 3.2), and students were assessed by a peer referencing system. Grades awarded reflected the position of the student within Sweden and a set of correction factors were applied to ensure that the grades were equivalent between schools. Using this system, the grades had minimal grade inflation over time and were normally distributed. From 1998, the score was expressed on scale between 10 (lowest) and 320 (overall mean was 207) utilizing a criterion referenced system, in which students were assessed for their achievement of certain competencies. Scores were not standardized across schools or constructed to produce a normal distribution.
Educational attainment (EA)	Longitudinal integrated database for health insurance and labour market studies (LISA)	Number of years of education are measured in 7 different levels:<br>1 Pre-high school < 9 years<br>2 High School 9 years<br>3 Upper Secondary School < 3 years<br>4 Upper Secondary School 3 years<br>5 Post-secondary education < 3years<br>6 Post-secondary education 3 years or more<br>7 Research education (PhD).
Divorce	Register of Total Population	Divorce is defined as the first divorce during our follow up period. In the models we use it as a binary variable.
Marriage	Register of Total Population	Marriage is defined as the first marriage during our follow up period. In the models we use it as a binary variable.
Anxiety Disorder (AD)	The Swedish Hospital Discharge Register (coverage 1973-2018); Outpatient Care Register (national coverage 2001-2018); Primary Care Registry (Partly coverage from 1999-2018)	ICD-8: 300.0, 300.2<br>ICD-9: 300A, 300C<br>ICD-10: F40, F41
Attention deficit hyperactivity disorder (ADHD)	The Swedish Hospital Discharge Register (coverage 1973-2018); Outpatient Care Register (national coverage 2001-2018); Primary Care Registry (Partly coverage from 1999-2018)	ICD-9: 314<br>ICD-10: F90

Bipolar Disorder (BD)	The Swedish Hospital Discharge Register (coverage 1973-2018); Outpatient Care Register (national coverage 2001-2018); Primary Care Registry (Partly coverage from 1999-2018).	ICD-8: 296.1, 296.3, 296.8, 296.9, 298.1; ICD-9: 296A, 296C, 296D, 296E, 296W, 298B; ICD-10: F30, F31
Drug Use Disorder (DUD)	The Swedish Hospital Discharge Register (coverage 1973-2018); Outpatient Care Register (national coverage 2001-2018); Primary Care Registry (Partly coverage from 1999-2018); the Swedish Drug Register (2005-2018); the Swedish Mortality Register, and the Swedish Criminal Register (1973-2018) and the Swedish Suspicion Register (1998-2018)	Drug abuse (DA) was identified in the Swedish medical and mortality registries by ICD codes (ICD-8: Drug dependence (304); ICD-9: Drug psychoses (292) and Drug dependence (304); ICD-10: Mental and behavioral disorders due to psychoactive substance use (F10-F19), except those due to alcohol (F10) or tobacco (F17)); in the Suspicion Register by codes 3070, 5010, 5011, and 5012, that reflect crimes related to DA; and in the Crime Register by references to laws covering narcotics (law 1968:64, paragraph 1, point 6) and drug-related driving offences (law 1951:649, paragraph 4, subsection 2 and paragraph 4A, subsection 2). DA was identified in individuals (excluding those suffering from cancer) in the Prescribed Drug Register who had retrieved (in average) more than four defined daily doses a day for 12 months from either of Hypnotics and Sedatives (Anatomical Therapeutic Chemical (ATC) Classification System N05C and N05BA) or Opioids (ATC: N02A).
Major Depression (MD)	The Swedish Hospital Discharge Register (coverage 1973-2018); Outpatient Care Register (national coverage 2001-2018); Primary Care Registry (Partly coverage from 1999-2018)	ICD-8: 296.2, 298.0, 300.4<br>ICD-9: 296.2, 296.4, 298.0, 300.4<br>ICD-10: F32, F33.
PTSD	The Swedish Hospital Discharge Register (coverage 1973-2017); Outpatient Care Register (national coverage 2001-2017); Primary Care Registry (Partly coverage from 1999-2017)	ICD-8 and ICD-9: 308, 309<br>ICD-10 F43; F630
Resilience (RES)	Military Conscription Register	Resilience was designed by the Swedish military to assess the ability to cope with psychologically stressful situations and was scored on a normally distributed 1–9 scale. Psychologists, who conduct a semi-structured interview averaging 25 minutes with every conscript, assign the score. The conscript is encouraged to describe his everyday life, covering five areas: school, work experiences, leisure time, home environment, and emotional stability. Background information including school grades, job experiences, and test results is provided to the interviewer in advance. Note that this measure is only for males.
Area Deprivation (Dep)	Longitudinal integrated database for health insurance and labor market studies (LISA), Register of Total Population	For every year an individual is registered at a specific DeSO area. The DeSO areas divides Sweden into 5,983 areas and have between 700 and 2,700 inhabitants. The division considers the geographical conditions so that the boundaries follow, for example, streets, waterways and railways. Important building blocks used to create DeSO are urban areas and electoral districts. For each of the DeSO area we created a neighborhood social deprivation (SD) index based on register data for all residents in the neighborhood aged 25-64. We used deprivation indicators used by past studies to characterize neighborhood environments and then used a principal component analysis to calculate a z-score. The following four variables were included: low educational status (defined as less than 10 years of formal education); low income (from all sources, including that from interest and dividends, which was defined as less than 50% of individual median income); unemployment (defined as not employed; excluding full-time students, those completing compulsory military service, and early retirees); and social welfare assistance. We define high deprivation as above one standard deviation from the mean value. The variable was measured during ages 30 to 34 of the individual. We divided the variable into three groups: 0: no years residing in deprived area; 1: 1 year residing in deprived area; 2: more than 1 year residing in deprived area.
Early Retirement (ER)	Longitudinal integrated database for health insurance and labour market studies (LISA)	Early retirement is a combination of several different variables from the Swedish registers. Until year 2002, Early Retirement Pension was paid to people aged 16-64 and granted when their working capability was deemed to be permanently reduced by at least one quarter due to medical reasons. The early retirement variable is also composed of temporary disability pensions paid to individuals whose working capability was not expected to be permanent but was expected to persist for a considerable time. From 2003, the rules for Early Retirement Pension and Temporary disability pension changed and

		these types of compensations were changed into sickness compensation (for individuals 30-64) and activity compensation (for individuals 19-29). The qualification rules were similar but activity compensation was supposed to be limited in time. We consider an individual as a recipient of early retirement if she/he has any registrations of early retirement during a year. The variable was measured during ages 30 to 34 of the individual. We divided the variable into three groups: 0: no early retirement; 1: 1 year with early retirement; 2: more than 1 year with early retirement
Sick leave (SL)	Longitudinal integrated database for health insurance and labour market studies (LISA)	During the first 14 days of sick leave, the employers are responsible for sick pay. Because this pay cannot be differentiated from ordinary salary, Statistics Sweden cannot identify sick leave ≤ 14 days. If a sick leave episode is ≤ 14 days, the episode does not enter into the sick leave statistics in LISA. We consider an individual as a recipient of sick leave benefits if she/he has any registrations of sick leave benefits during a year. The variable was measured during ages 30 to 34 of the individual. We divided the variable into three groups: 0: no sick leave; 1: 1 year with sick leave; 2: more than 1 year with sick leave
Social welfare recipient (SW)	Longitudinal integrated database for health insurance and labour market studies (LISA)	Social assistance is defined as financial support under the Social Services Act. You can receive support for your upkeep and for other items that you need to have a reasonable standard of living. Examples of common situations when social assistance is given: As an income supplement to low-income families; for unemployed when other unemployment assistance is not provided or is insufficient; when sickness benefits are insufficient or not provided; to those who are bound by the children in the home and cannot get childcare and therefore not can seek work. The variable is recorded at the family level, which means that all individuals in a family with social assistance will, in this report, be counted as recipients of social welfare. The variable was measured during ages 30 to 34 of the individual. We consider an individual as a recipient of social welfare if she/he has any registrations of social welfare during a year. We divided the variable into three groups: 0: no social welfare; 1: 1 year with social welfare; 2: more than 1 year with social welfare
Unemployment status (UE)	Longitudinal integrated database for health insurance and labour market studies (LISA)	In order to be entitled to unemployment benefits the following requirements needs to be fulfilled. The individual is: unemployed (fully or partially); able to work at least 3 hours a day and 17 hours a week; registered as a jobseeker at the Employment Service; prepared to take the offer of suitable work or employment; actively seeking for a suitable job.<br>We consider an individual as unemployed during a year if she/he has more than 10 days during a year with unemployment benefits registered. The variable was measured during ages 30 to 34 of the individual. We divided the variable into three groups: 0: no unemployment benefits; 1: 1 year with unemployment benefits; 2: more than 1 year with unemployment benefits

Supplementary Table 2. Description of process used in the calculation of family genetic risk scores.

The dataset for the calculations includes:<br>Column1 = Identification number of the proband (Born 1932-1995)<br>Column2 = Identification number of the relative (1st to 5th degree relatives)<br>Column3 = Proportion of shared additive genetic effects (0.03125 to 0.50) with the proband<br>Column4 = Year of Birth of relative<br>Column5 = Sex of relative<br>Column6 = Age at registration for trait<br>Column7 = Age at end of follow-up (2018-12-31 or age at death, or age at emigration whichever came first)
Step 1: Using all unique relatives with a registration for the disorder, we non-parametrically estimated the distribution of age at first registration. The empirical distribution is used to obtain weights for relatives without a registration for the disorder, to account for the proportion of the time-at-risk period they had completed at the end of follow-up. For example, for relatives at age x at end of follow-up, the weight corresponds to the proportion of relatives registered for the trait that had been registration at age x. For relatives born prior to 1958 we subtracted age at the end of follow-up with the following formula: 1958 - Year of birth of relative. The range of birth years for relatives included in these analyses was 1892-2018. This modification was done to control for registration effects (i.e., most registers in Sweden start in 1973, suggesting that relatives from early birth cohorts do not have the possibility to be registered at younger ages). Note that all relatives with the disorder are weighted one.
Step 2: Transform the binary variable (trait yes/no) into a z-score based on the threshold for each trait. The underlying liability of the individual is not assessable. Instead, we estimated the mean of the underlying liability to obtain sex and birth decade specific Z-scores for relatives with the trait registration and relatives without the trait. We generate n random numbers from a N (0, 1) distribution and estimate the mean for relatives registered with the disorder (i.e., mean of the observations above the threshold) and for relatives without a registration (i.e., mean of all observation below the threshold). The thresholds are calculated for each decade of birth and sex.
Step 3: Correct for cohabitation effects. To estimate the cohabitation effect (i.e. “shared environment”), we created a database with all individuals in the Swedish population born in Sweden 1955-1990. We also included the number of years, during ages 0-15, that individuals resided in the same household as their biological father. We thereby were able to define two kinds of families i) “not-lived-with” father families (offspring never resided for more than 1 year in the same household or in the same community as their biological father); ii) “lived-with” father (offspring resided a minimum of 13 year in the same household as their biological father. We performed a logistic regression model with the binary trait in offspring as outcome and the binary trait in father, type of father, and their interaction as predictors. We used the interaction term as the difference of effect between genes only and genes + environment. The same approach was performed for half-siblings where we compared those who were reared together versus reared apart. The following interaction terms were used in the calculations for each of our disorders:<br><br>(inner table: columns: blank / Parent/Children / Siblings; SA: 0.66 / 0.76; AUD: 0.99 / 0.69)
Step 4: Calculate the product for each relative using the four components:<br>i) Z-score (reflecting sex and year of birth adjusted rates)<br>ii) Weight (reflecting the proportion of risk period they had completed)<br>iii) Cohabitation effects<br>iv) Proportion of shared genetic effects (0.03125 – 0.5) with the proband
Step 5: Average the product calculated in step 4 across all relatives to a proband
Step 6: Correct for the number of relatives. We multiplied the results from step 5 with a shrinkage factor. Shrinkage factor (SF): B/(B+A/C). It produces more shrinkage if B and C are small and A is large.<br>(A) the variance of the z-score of the disorder across all relatives,<br>(B) the variance in the mean z-score across all probands,<br>(C) the weighted number of relatives for each proband (sum of Column 3 across each proband).
Step 7: Correct for difference by year of birth and county differences. There are 21 counties in Sweden. For each proband we used the county they had resided in during the maximum number of years (measured from 1969 and onwards) We standardized the risk score by year of birth and county of the proband into a z-score with mean 0 and SD 1.

	Parent/Children	Siblings
SA	0.66	0.76
AUD	0.99	0.69

Supplementary Table 3. Correlations and 95% confidence intervals between family genetic risk scores (FGRS) for alcohol use disorder (AUD) and suicide attempt (SA) and each outcome variable. For binary outcomes, point biserial correlations are reported; for continuous outcomes, Pearson correlations are reported.

**Domain**	**Phenotype**	**$FGRS_{AUD}$**	**$FGRS_{SA}$**
n/a	Alcohol use disorder	0.136 (0.135; 0.138)	0.084 (0.083; 0.086)
n/a	Suicide attempt	0.083 (0.081; 0.084)	0.097 (0.096; 0.098)
Education	Academic achievement	-0.211 (-0.212; -0.209)	-0.149 (-0.151; -0.148)
	Educational attainment	-0.165 (-0.166; -0.164)	-0.111 (-0.111; -0.112)
Family	Divorce	0.045 (0.043; 0.047)	0.036 (0.035; 0.038)
	Married	-0.035 (-0.037; -0.034)	-0.011 (-0.012; -0.001)
Psychiatric	Anxiety disorders	0.088 (0.086; 0.089)	0.068 (0.067; 0.070)
	Attention deficit hyperactivity disorder	0.068 (0.067; 0.069)	0.053 (0.052; 0.055)
	Bipolar Disorder	0.039 (0.037; 0.040)	0.038 (0.036; 0.039)
	Drug use disorder	0.132 (0.131; 0.134)	0.090 (0.089; 0.092)
	Major depression	0.083 (0.082; 0.085)	0.072 (0.071; 0.073)
	Posttraumatic stress disorder	0.061 (0.059; 0.062)	0.056 (0.054; 0.057)
	Resilience	-0122 (-0.124; -0.120)	-0.081 (-0.083; -0.079)
Socioeconomic	Area Deprivation	0.070 (0.069; 0.072)	0.053 (0.051; 0.054)
	Early Retirement	0.050 (0.049; 0.052)	0.045 (0.044; 0.047)
	Sick Leave	0.064 (0.062; 0.065)	0.057 (0.056; 0.059)
	Social Welfare	0.140 (0.138; 0.141)	0.105 (0.104; 0.107)
	Unemployment	0.083 (0.082; 0.085)	0.057 (0.056; 0.059)

Supplementary Figure captions

Supplementary Figure 1. Correlations from sensitivity analyses in which all individuals with alcohol use disorder or suicide attempt registrations were excluded. Colors reflect the domain of inquiry (blue = psychiatric/psychological outcomes; red = socioeconomic outcomes; green = family outcomes; grey = education outcomes). The darker color represents the correlation from the primary analysis with no exclusions, while the lighter color represents the correlation from the sensitivity analysis. *Notes for Supplementary Figure 1: AA=academic achievement; EA=educational attainment; DIV=divorce; MAR=marriage; AD=anxiety disorders; ADHD=attention deficit hyperactivity disorder; BD=bipolar disorder; DUD=drug use disorder; MD=major depression; PTSD=post-traumatic stress disorder; RES=resilience; DEP=deprived neighborhood residence; ER=early retirement; SL=sick leave; SW=social welfare; UE=unemployment*

Supplementary Figure 2. Comparison of correlations (with 95% confidence intervals) from the Low AUD_G/High SA_G group with correlations from a *post hoc* analysis group, in which the test subsample had low $FGRS_{AUD}$ and median $FGRS_{SA}$, and the reference subsample had median FGRS for both conditions. Rows represent thematic categories; columns represent the two groups. Within row, the y-axis scale is consistent across groups, and the y-axis varies across rows. *Notes for Supplementary Figure 2: AA=academic achievement; EA=educational attainment; DIV=divorce; MAR=marriage; AD=anxiety disorders; ADHD=attention deficit hyperactivity disorder; BD=bipolar disorder; DUD=drug use disorder; MD=major depression; PTSD=post-traumatic stress disorder; RES=resilience; DEP=deprived neighborhood residence; ER=early retirement; SL=sick leave; SW=social welfare; UE=unemployment*

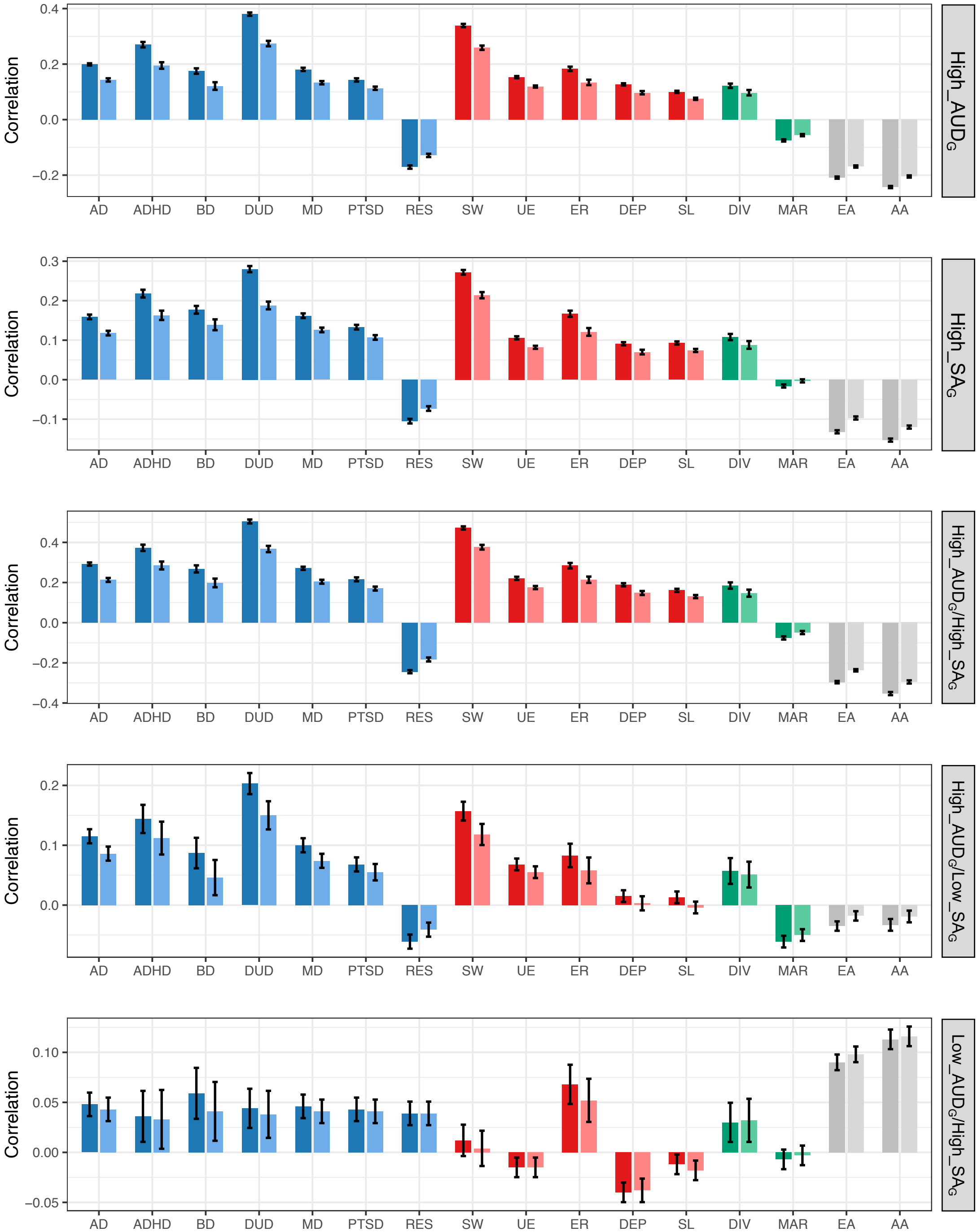
Correlation
AD
ADHD
BD
DUD
MD
PTSD
RES
SW
UE
ER
DEP
SL
DIV
MAR
EA
AA
High_AUDG
High_SAG
High_AUDG/High_SAG
High_AUDG/Low_SAG
Low_AUDG/High_SAG

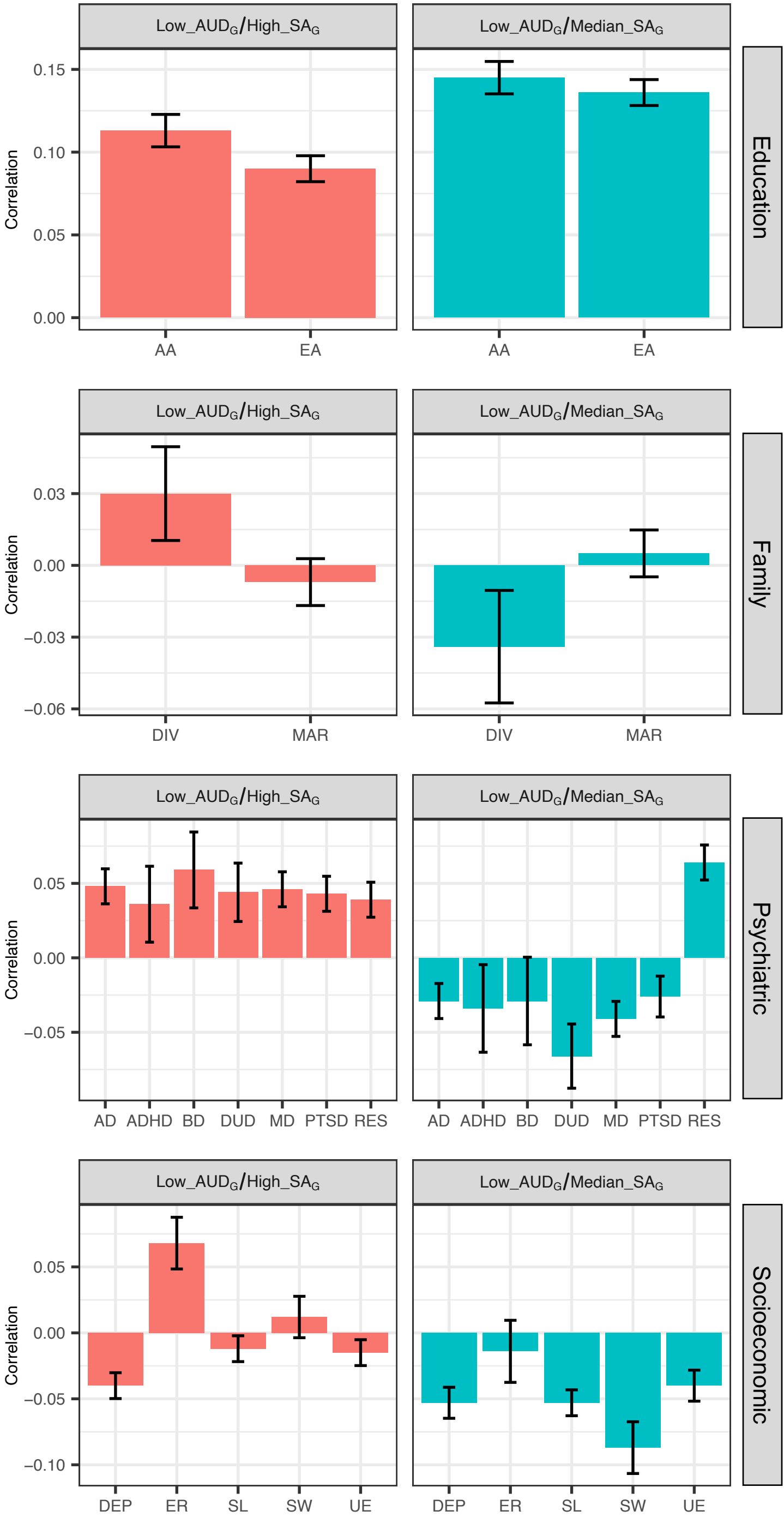
Low_AUDG/High_SAG
Low_AUDG/Median_SAG
Education
Family
Psychiatric
Socioeconomic
Correlation
0.15
0.10
0.05
0.00
0.03
−0.03
−0.06
−0.05
−0.10
AA
EA
DIV
MAR
AD
ADHD
BD
DUD
MD
PTSD
RES
DEP
ER
SL
SW
UE
